# Case report: Regression of Glioblastoma after flavivirus infection

**DOI:** 10.3389/fmed.2023.1192070

**Published:** 2023-06-01

**Authors:** Patricia P. Garcez, André Guasti, Nina Ventura, Luiza Mendonça Higa, Felipe Andreiuolo, Gabriella Pinheiro A. de Freitas, Liane de Jesus Ribeiro, Richard Araújo Maia, Sheila Maria Barbosa de Lima, Adriana de Souza Azevedo, Waleska Dias Schwarcz, Elena Cristina Caride, Leila Chimelli, Luiz Gustavo Dubois, Orlando da Costa Ferreira Júnior, Amilcar Tanuri, Vivaldo Moura-Neto, Paulo Niemeyer

**Affiliations:** ^1^Instituto de Ciências Biomédicas, Universidade Federal do Rio de Janeiro, Rio de Janeiro, Brazil; ^2^Instituto Estadual do Cérebro Paulo Niemeyer, Rio de Janeiro, Brazil; ^3^Hospital Federal de Bonsucesso, Rio de Janeiro, Brazil; ^4^Departamento de Radiologia, Universidade Federal do Rio de Janeiro, Rio de Janeiro, Brazil; ^5^Instituto de Biologia, Universidade Federal do Rio de Janeiro, Rio de Janeiro, Brazil; ^6^Núcleo de Enfrentamentos e Estudos de Doenças Infecciosas Emergentes e Reemergentes (NEEDIER), Universidade Federal do Rio de Janeiro, Rio de Janeiro, Brazil; ^7^Instituto de Tecnologia em Imunobiológicos (Bio-Manguinhos), Fundação Oswaldo Cruz, Rio de Janeiro, Brazil; ^8^Campus UFRJ Duque de Caxias Prof. Geraldo Cidade, Universidade Federal do Rio de Janeiro, Rio de Janeiro, Brazil

**Keywords:** glioblastoma, flavivirus, oncolytic virus, ZIKV, DENV, immunovirotherapy

## Abstract

Glioblastoma is the most frequent and aggressive primary brain cancer. In preclinical studies, Zika virus, a flavivirus that triggers the death of glioblastoma stem-like cells. However, the flavivirus oncolytic activity has not been demonstrated in human patients. Here we report a glioblastoma patient who received the standard of care therapy, including surgical resection, radiotherapy and temozolomide. However, shortly after the tumor mass resection, the patient was clinically diagnosed with a typical arbovirus-like infection, during a Zika virus outbreak in Brazil. Following the infection resolution, the glioblastoma regressed, and no recurrence was observed. This clinical response continues 6 years after the glioblastoma initial diagnosis.

## Introduction

Glioblastoma is an intracerebral tumor with glial origin with the highest prevalence and malignancy. It exhibits a high proliferative and migratory profile with aggressive invasive ability. The current treatment consists of surgery associated with radio and chemotherapy. However, glioblastoma is endowed with a subpopulation of cancer stem-like cells, which confers to this tumor high heterogeneity and resistance to multiple drugs, in general, re-growing a few months after surgery. Therefore, the patient's life expectancy after diagnosis is, on average, 15 months (1).

Glioblastoma treatment is considered a scientific and public health challenge. Therefore, it is urgent to implement innovative research to produce confident results regarding understanding glioblastoma pathophysiology, establishing precise diagnostic tools and efficient alternative treatment.

Recently, multiple scientific studies demonstrated that Zika virus (ZIKV), a virus from the Flaviviridae family, related to Dengue virus (DENV), Yellow fever and West Nile viruses, specifically infect and induce apoptosis in glioblastoma stem-like cells *in vitro* and *in vivo* using human cells, human explants, and animal models (2, 3). It is now known that ZIKV binds to the Integrin α_v_β_5_ receptor expressed in the glioblastoma stem-like cells (3). As foreseen in other oncolytic viral studies (4), ZIKV infection also recruits T cells to the tumor microenvironment contributing to its reduction (5, 6). Furthermore, ZIKV intracranial administration protected against subsequent tumor challenges by eliciting an anti-tumor memory response in mice (5, 6). ZIKV infection also improved the symptoms and increased survival of animals bearing other nervous system tumors such as oligodendroglioma, meningioma and medulloblastoma (7, 8). Therefore, to take advantage of ZIKV tropism and oncolytic ability to treat this heterogeneous and drug-resistant tumor is an exciting perspective.

Here we present a case report of one patient diagnosed with glioblastoma and, after the tumor resection, was infected with an arbovirus-like infection.

## Case report

**Figure 1:**
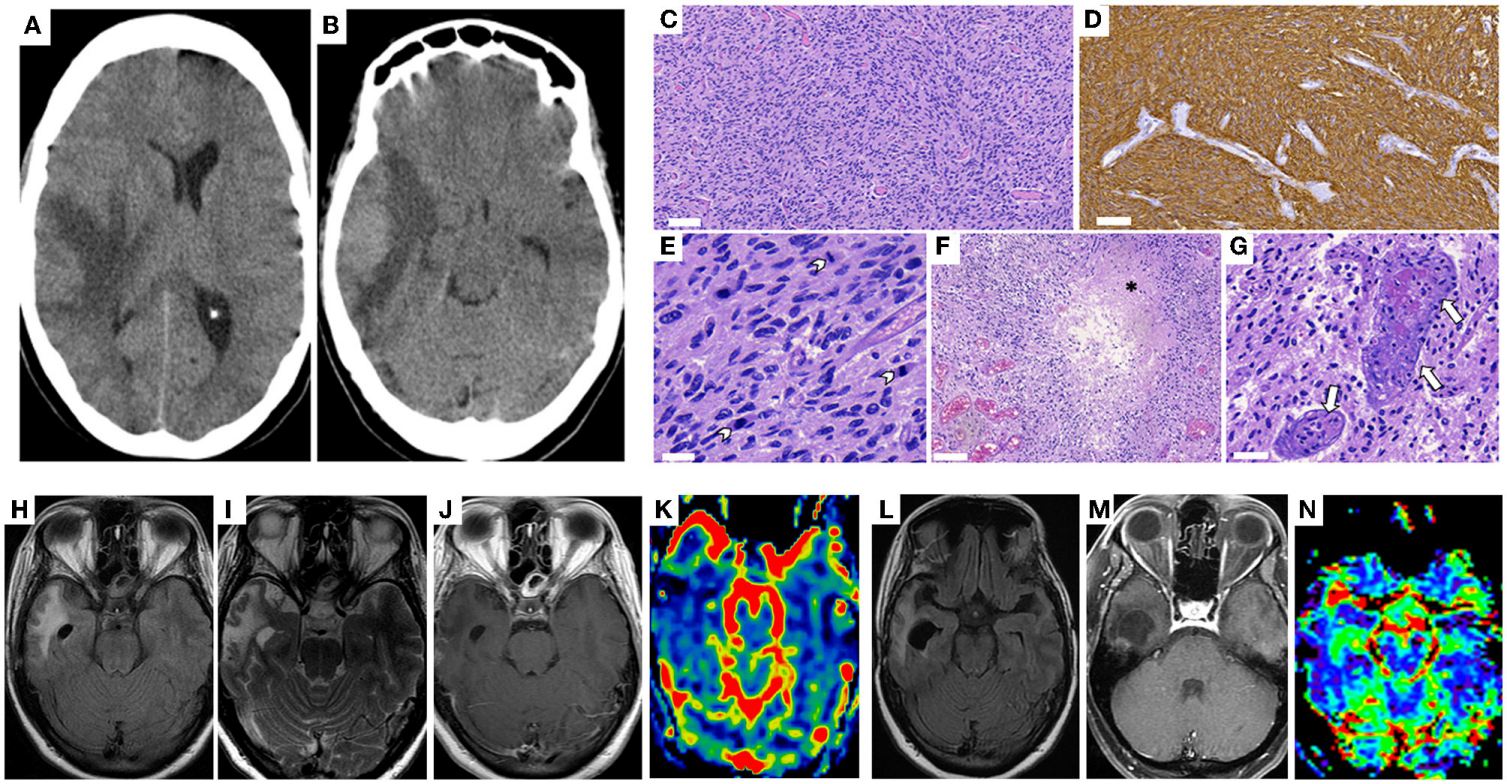
**(A, B)** Non-contrast computed tomography imaging in April 2016 before the resection surgery. It demonstrates an expansive lesion on the right temporal lobe **(A)**, associated with moderate oedema and compression of surrounding structures **(B)**, including left deviation of the midline. **(C–G)** Histopathological analysis of tumor sample. Sections were stained with H&E **(C)** and display high cellularity. Scale bar: 100 mm. **(D)** Immunostaining for GFAP showing abundance of glial marker. Scale bar: 60 mm. **(E)** Sections were stained with H&E and arrowheads show examples of mitotic figures. Scale bar: 30 mm. **(F)** Using H&E staining, asterisk indicate the tumor necrotic center. Scale bar: 100 mm. **(G)** Using H&E staining, arrows point to abnormal microvascular proliferation. Scale bar: 40 mm. **(H–K)** MRI six months after the resection surgery in October 2016. Axial FLAIR **(H)**, Axial T2-weighted **(I)**, and axial post-contrast T1-weighted **(J)** images demonstrate surgical manipulation on the right temporal lobe. No enhancing or expansive lesions are seen. Axial r-CBV map **(K)** does not show areas of hyperperfusion. **(L–N)** MRI in May 2022. Axial FLAIR **(L)**, and axial post-contrast T1-weighted images **(M)** demonstrate surgical manipulation on the right temporal lobe. No enhancing or expansive lesions are seen. Axial r-CBV map **(N)** does not show areas of hyperperfusion.

**Figure 2:**
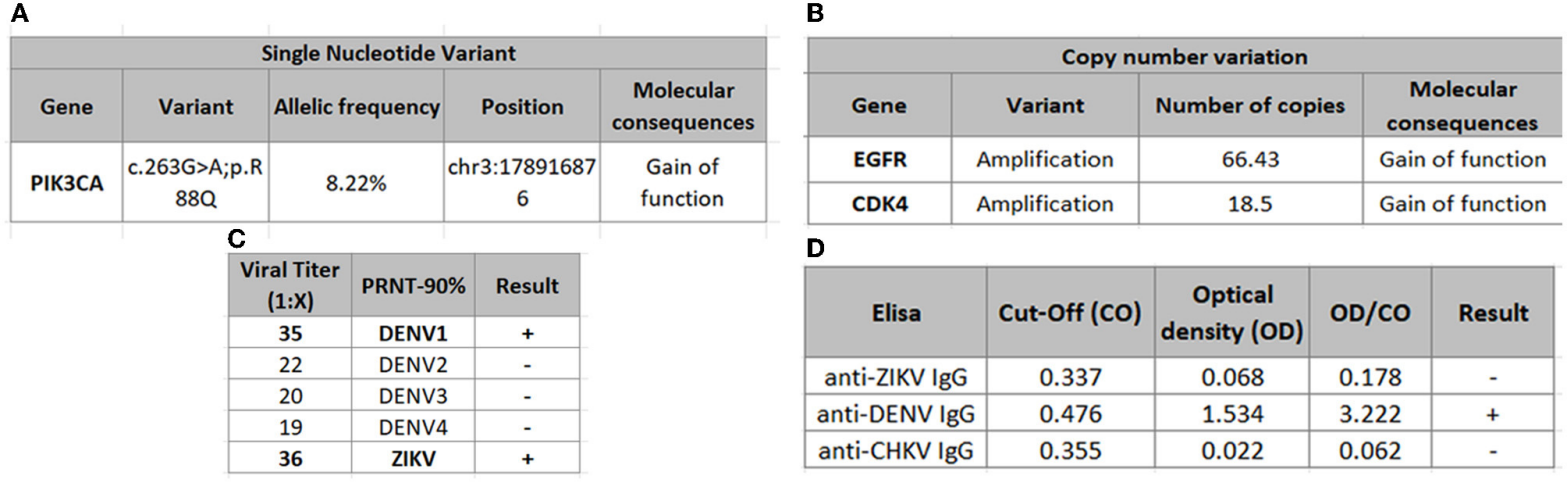
**(A, B)** Genetic alterations in Next Generation Sequencing. IDH1 and IDH2 genes had no Single Nucleotide Variation detected. **(C)** Positive neutralizing antibodies against ZIKV and DENV1. DENV (four serotypes) and ZIKV were tested with the patient's serum samples. Titers were calculated using the serum ability to reduce 90% of plaque number, compared to controls (using different virus). The cut-off used is 30. DENV1 and ZIKV presented similar neutralizing antibodies titers. Because of flavivirus classic cross-reaction, it is possible to conclude that the patient has flavivirus neutralizing antibodies. **(D)** Positive IgG antibodies against DENV detected by MAC-ELISA.

The 43-year-old woman suffered a fall at home in April 2016. Imaging identified an expansive lesion associated with edema in the right temporal lobe, causing compression and midline deviation suggesting a brain tumor (Figures 1A, B). She was taken to surgery on the 18th of April 2016, when a right temporal craniotomy was performed. The macroscopic appearance of the lesion was typical of an infiltrative high-grade glioma. The lesion had no cleavage plane with the brain parenchyma, and necrotic areas were observed. A wide resection of the lesion was possible, and the tumor was excised. Histopathology confirmed the tumor as glioblastoma (Figures 1C–G). High Throughput Sequencing identified that the tumor bears wild-type copies for *IDH1* and *IDH2* with mutations in oncogenes such as PIK3CA, clinically associated with patients who develop glioblastoma at a young age and poor survival prognosis (9) (Figures 2A, B). The patient has received standard-of-care therapy after surgery, with radiotherapy and chemotherapy with temozolomide. Three and a half weeks after the tumor resection in 2016, which coincided with the Zika virus pandemic, the patient was clinically diagnosed with an arbovirus-like infection. The infection has manifested as low-grade fever, a robust cutaneous rash, arthralgia, and potent myalgia, lasting for seven days. Antibody detection was performed using neutralizing antibody assay (PRNT), and IgM antibody capture enzyme-linked immunosorbent assay (ELISA). The PRNT resulted in a cross-reaction between DENV and ZIKV antibodies (Figure 2C). In contrast, ELISA revealed that the patient is positive for DENV and negative for ZIKV and CHIKV IgG antibodies (Figure 2D). Patient's serum sample presented similar neutralizing antibody titers against DENV1 and ZIKV and it was considered positive for both viruses. The is a flavivirus classic cross-reaction, therefore it is possible to conclude that the patient has specific flavivirus neutralizing antibodies. These analyses were performed 3 years after the related infection.

Six months after the surgery, the brain's magnetic resonance imaging (MRI) showed no evidence of tumor recurrence (Figures 1H–K). After that, the patient was followed-up with brain scans regularly. Two years and five months after the tumor resection, the patient was diagnosed with a brain aneurysm on the left hemisphere, contralateral to the previous glioblastoma lesion, and submitted to a microsurgical clipping (Supplementary Figures 1, 2). Since then, the patient has been followed up with MRI every 6 months without tumor recurrence (Figures 1L–N). She is asymptomatic, actively working, and does not use medication such as anticonvulsants.

## Discussion

Recent reports have shown that flavivirus has oncolytic activity, eliminating tumor stem-like cells, up-regulating memory T-cells, and leading to tumor depletion *in vivo* (2, 3, 5, 10). Here, we describe a clinical case of a patient who, shortly after the glioblastoma surgical resection, has been naturally infected with a typical arbovirus-like infection. Since then, more than seven years later, the patient had no glioblastoma recurrence. Although the patient has had a clinical diagnosis of ZIKV based on her clinical signs and the epidemiological context, the neutralizing antibody assay results revealed a cross-reaction between ZIKV and DENV and ELISA confirmed that the patient has IgG antibodies against DENV. As the samples were obtained 3 years after the viral infection diagnosis, we can only confirm that the patient had a flavivirus infection. It would have been beneficial to have blood samples and CSF from the glioblastoma patient during the acute and convalescent phases of the infection. First, using samples from the acute phase of infection would have been ideal for identifying which flavivirus caused the infection using RT-qPCR. Similarly, we could have confirmed that the virus had crossed the blood-brain barrier using the CSF samples from the acute phase. In addition, using sera or plasma samples from both the acute and the convalescent stages of infection, it would have been possible to identify the IgG seroconversion through ELISA and PRNT against the endemic arbovirus. Comparison between titers would have provided a more accurate diagnosis. Finally, serological analyses could have confirmed the immune system recruitment, activation, and the presence of released inflammatory mediators.

In 2016, when the patient was clinically diagnosed with a flavivirus infection, Brazil and Rio de Janeiro faced an unprecedented ZIKV outbreak, whereas DENV is endemic in this region (11). In the same year, the WHO declared a public health emergency of international concern, because of the association between ZIKV and severe congenital malformations, such as microcephaly (12). As the investigation of the mechanisms of ZIKV infection during brain development progressed, many human cell lines were used as models, including glioblastoma cell lines. Surprisingly, ZIKV has shown oncolytic capacity, able to infect and trigger cell death mainly in the glioblastoma stem-like cell populations (Sox2+Ki67+) *in vitro* and *in vivo*, using animal models (2). However, the oncolytic activity of DENV against glioblastoma cells is still under investigation. Although Zika virus can infect blood vessels during brain development and cause endothelial dysfunction *in vivo* (13), it is unknown whether flavivirus infection could be related to vascular aneurysm. Altogether, flavivirus oncolytic ability could be explored as a novel brain cancer therapy to reduce the glioblastoma stem-like cells and therefore prolong the patient's lifespan.

In conclusion, the current case report provides valuable information for future studies investigating common flavivirus properties as a target for immunovirotherapy to potentially treat glioblastoma patients.

## Methods

### Imaging

Pre-operative non-contrast CT imaging was obtained with a 16-channel multidetector scanner, and post-operative MR images were obtained with a 1,5T scanner (Avanto, Siemens, Erlangen, Germany). The following sequences were routinely acquired: axial Fluid attenuated inversion recovery (FLAIR), axial T2-weighted images, pre and post-contrast T1-weighted images, post-contrast tridimensional T1-weighted images, susceptibility weighted sequences, diffusion weighted sequences, and dynamic susceptibility contrast-enhanced sequences (DSC). Single voxel spectroscopy of the surgical manipulated area was obtained in some exams.

### Immunostaining

Tumor blocks were fixed with 4% paraformaldehyde (Sigma-Aldrich, USA) in phosphate-buffered saline for 15 min at 37°C, mounted in paraffin. Thin sections (5 μm) were obtained with a microtome (Leica, Germany), permeabilized with 0.3% Triton X-100 (Sigma-Aldrich, USA), incubated in 50 mM ammonium chloride, followed by 3% bovine serum albumin blocking (Sigma-Aldrich, USA). Incubation with rabbit anti-human-Sox2 (1:100; Merck-Millipore, Germany), rabbit anti-Vimentin (1:500, abcam, USA) and mouse anti-phospho (S55) Vimentin (1:250, abcam, USA) was performed overnight. Subsequently, samples were incubated with secondary antibodies: goat anti-rabbit Alexa Fluor 488 IgG (1:400; Thermo Fisher Scientific, USA) and goat anti-mouse Alexa Fluor 594 IgG (1:400; Thermo Fisher Scientific, USA). Nuclei were stained with 0.5 μg/mL 4′-6- diamino-2-phenylindole (DAPI) for 5 min. Images were acquired with TCS SP8 confocal microscope (Leica, Germany) with an oil immersion 20x objective, (0.75 NA).

### ELISA

Antibodies against ZIKV, DENV and CHIKV (IgG) were investigated using ELISA (Euroimmun). Serum samples were processed according to the manufacturer's instructions. Positive samples presented optical density/cutoff ratio (OD/CO) equal to or higher than 1.

### Plaque reduction neutralization test (PRNT)

The PRNT was performed to quantify neutralizing antibodies in the serum sample of the patient. Serial dilutions of the serum sample were incubated with approximately 100 PFU of ZIKV (ES 2916/201) or DENV1-4 (DENV1 60305; DENV2 44/12; DENV3 16562; DENV4 TVP 360) for 1 h at 37°C. The serum–virus mixture was inoculated into confluent Vero CCL-81 (ATCC) monolayers. After 1 h, the inoculum was removed, and semisolid medium (2%carboxymethylcellulose in 199 medium supplemented with 5% fetal bovine serum) was added. The plates were further incubated for 4–8 days and then fixed with formaldehyde solution, stained with crystal violet and wells were photographed with the Biospot^®^ and plaques were counted manually. Neutralizing antibody titers were expressed by PRNT_90%_ as the reciprocal of the serum dilution able to neutralize the viral infection by 90%. Seropositivity was determined considering a serum dilution higher than 1:30.

### Sequencing

DNA and RNA were extracted from the tumor paraffin block, according to the manufacturer's instructions. Libraries were built using Thermo Fisher kit. NGS (Next-Generation Sequencing) was performed using the Ion Torrent S5 (Thermo Fisher) Oncomine Focus platform associated with the Ampliseq customized panel. Hotspots, SNVs, indels, and CNVs variants are identified with Ion Reporter software and Oncomine Knowledgebase Assay platform using the GRCh37/hg19 genome as a reference.

## Data availability statement

The original contributions presented in the study are included in the article/Supplementary material, further inquiries can be directed to the corresponding authors.

## Ethics statement

Written informed consent was obtained from the patient for the publication of any potentially identifiable images or data included in this article. All protocols and procedures were approved by the Institutional Research Ethics Committee of Instituto Estadual do Cérebro Paulo Niemeyer under approved protocol 5.503.889. All experiments were performed in accordance with relevant guidelines and regulations. Written informed consent was obtained from the participant/patient(s) for the publication of this case report.

## Author contributions

PG: conceived the study and wrote the manuscript. AG: patient's neurosurgeon. NV: responsible for the imaging. LH: performed and analyzed the PRNT. FA: performed the histology analysis. GF: interviewed the patient. LR, RM, and AS: performed the immunology experiments. SL and WS: performed the PRNT. EC: coordinated the PRNT experiments and provided laboratory facility. LC: coordinated the neuropathology experiment and provided laboratory facility. LD: coordinated the sequencing experiment. OF: coordinated the immunology analysis. AT and VM-N: provided the laboratory facility. PN: provided the clinical facilities. All authors contributed to the article and approved the submitted version.
