# Supplementary material for: Case report: Regression of Glioblastoma after flavivirus infection
Source: Front Med (Lausanne). 2023 Jun 1;10:1192070. doi: 10.3389/fmed.2023.1192070 (PMC10267364; doi:10.3389/fmed.2023.1192070)
Supplement: Supplementary file 1 [file Data_Sheet_1.PDF]

Supplementary Material

Case Report: ***Regression of Glioblastoma after flavivirus infection***

Patricia P. Garcez[1*], André Guasti[2,3], Nina Ventura[2, 4], Luiza M. Higa[5], Felipe Andreiuolo[2], Gabriella Pinheiro A. Freitas[1], Adriana de Souza Azevedo Soares[6], Elena Cristina Caride[6], Leila Chimelli[2], Luiz Gustavo Dubois[2,7], Orlando da Costa Ferreira Júnior[5], Amilcar Tanuri[5], Vivaldo Moura-Neto[1,2], Paulo Niemeyer[2]

Supplementary Figure 1:

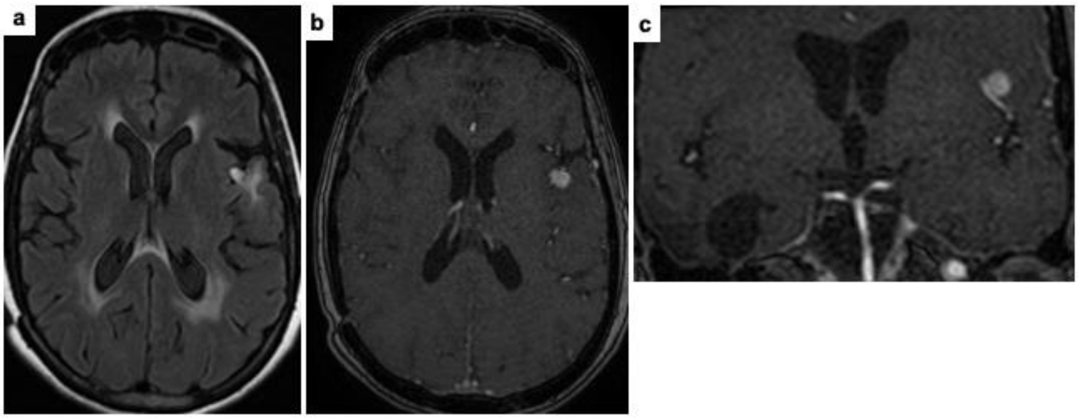

Brain MRI before surgical clipping in May 2019. Axial FLAIR (a), axial (b) and coronal (c) post-contrast Time of Flight sequence demonstrate a small, rounded lesion, with contrast enhancement, located in the left Sylvian fissure, in contact with M3 middle cerebral artery. FLAIR image (a) also demonstrates confluent periventricular white matter hyperintense areas, which could be related to radiotherapy.

Supplementary Figure 2:

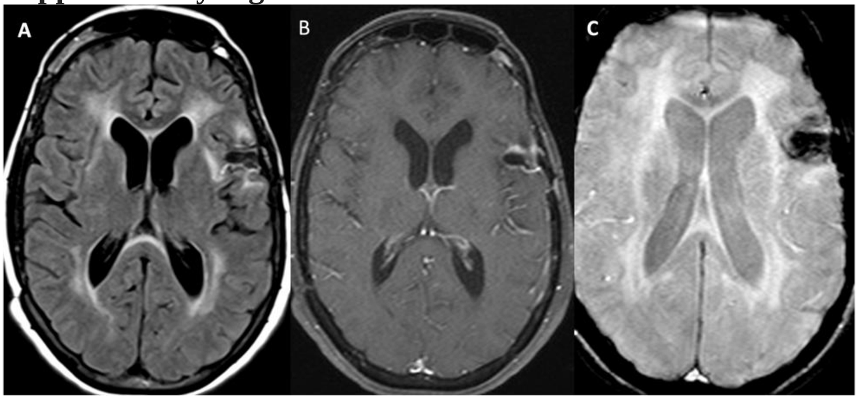

Brain MRI after surgical clipping in May 2019. Axial FLAIR (A), post-contrast axial T1-weighted (B) and susceptibility weighted (C) images demonstrate manipulation at the right inferior frontal gyrus/superior insula, with hemosiderin/ferritin deposits.
